# Disaster risk reduction: Integrating sustainable development goals and occupational safety and health in festival and event management

**DOI:** 10.4102/jamba.v14i1.1205

**Published:** 2022-08-10

**Authors:** Leonie B. Louw, Elriza Esterhuyzen

**Affiliations:** 1 Department of Operations Management, College of Economics and Management Sciences, University of South Africa, Pretoria, South Africa

**Keywords:** disaster management, disaster risk management, sustainability, OSH, OHS, safety, festivals, events

## Abstract

The purpose of this article is to develop a theoretical disaster risk reduction model, creating a virtuous cycle of knowledge and action across the festival and events industry, based on occupational safety and health (OSH) strategic objectives, as informed by a systematic literature review. The main constructs of this conceptual article are explored through a systematic literature review. Sources include publications of key stakeholders in the festival and event industry, applicable global directives, strategic documents of relevant governmental and non-governmental organisations and academic publications. From the data gathered in the systematic literature review it can be surmised that sustainable development goals (SDGs) related research in tourism, festivals, events and OSH is lacking in quantity and there is room for these aspects to be addressed in future research to ensure that these fields of study make a more substantial contribution to disaster risk reduction in festival and event management. This article is limited to secondary data collected through a systematic literature review, supported by additional literature to inform a theoretical framework incorporating SDGs, disaster risk reduction and OSH strategic objectives for festivals and events. Sustainable development goals are aimed at achieving a sustainable future for all. The detrimental effect of OSH incidents can be counterproductive to achieving such goals and should therefore be closely monitored and managed. Festival and event managers should thus take cognisance of the importance of OSH through a plan of action, benchmarked against best practice, to allow for enhanced disaster risk management. This article investigates the concepts of sustainability, disaster risk reduction, OSH, events and festival management and combines the concepts in a unique manner.

## Introduction

The African Union Commission (AUC) Agenda 2063 envisages a prosperous Africa based on inclusive growth and sustainable development, an Africa as an influential global partner and player (AUC 2015:2). The United Nations 2030 Agenda for Sustainable Development provides a plan of action aimed at sustainable development through collaborative partnerships between countries and stakeholders (UN 2015:1). The sustainable development goals (SDGs) allow for their application in various industries. The aim of this conceptual article is to integrate the fields of festival and event management with occupational safety and health (OSH) to illustrate how these two fields of study can contribute towards the achievement of the SDGs. This will be achieved by focusing specifically on SDG 3.9 (reducing the number deaths and illness from hazardous chemicals and air, water and soil pollution and contaminants); SDG 8.8 (protecting labour rights and promoting safe and secure working environments for all staff); and SDG 16.6 (developing effective, accountable and transparent institutions at all levels) (International Labour Organization [ILO] 2020b:1). Drawing from contemporary publications by the ILO, United Nations World Tourism Organization (UNWTO), National Institute of Occupational Health (NIOH) and academic, peer reviewed articles, the authors will conclude this article with the composition of a framework that festival and event managers can consider to enhance their effort in contributing towards the achievement of the identified SDGs.

## Methods

The main constructs of this conceptual article are explored through a systematic literature review. Sources include publications of key stakeholders (such as international governing bodies, government departments and trade industry publications) in the festival and event industry, applicable global directives, strategic documents of relevant governmental and non-governmental organisations and academic publications. The systematic literature review was conducted by utilising the Scopus database to inform a cross-sectional descriptive analysis of relevant scientific literature relating to the identified SDGs, festivals, events and OSH. The search results are summarised in Table 1.

**TABLE 1:** Summary of key terms and main constructs of Scopus search.

Key terms	Results	Main constructs
‘SDG 3’ *Ensure healthy lives and promote well-being for all at all ages*	141	AIDS (12) Air pollution (2) Biodiversity (1) Circular economy (2) Contraceptive (2) Dementia (1) Ebola (1) Education (3) Electricity consumption (1) Food security (3) Forest therapy (1) Gender inequality (11) Governance (22) Health systems (21) Healthy cities (7) Human rights (1) Infant and/or maternal mortality (30) Primary caregiver (2) Research (3) Stakeholder engagement (6)
‘SDG 3.9’ *Substantially reduce the number of deaths and illnesses from hazardous chemicals and air, water and soil pollution and contamination*	None	None
‘SDG 8’ *Promote sustained, inclusive and sustainable economic growth, full and productive employment and decent work for all*	44	Bio-economy (4) Circular economy (3) Economic growth (4) Environmental quality (2) Full employment (5) Global risk reduction (1) Health (3) SDG reporting (5) SME (1) Social economy (7) Supply chain (4) Tourism (3) Workplace culture (2)
‘SDG 8.8’ *Protect labour rights and promote safe and secure working environments for all workers, including migrant workers, in particular women migrants, and those in precarious employment*	8	Diet (1) Emergency care (1) Household energy (1) Gender equality (1) Natural resources (1) Land acquisition (1) Smoking (2)
‘SDG 16’ *Promote peaceful and inclusive societies for sustainable development, provide access to justice for all and build effective, accountable and inclusive institutions at all levels*	33	Anti-corruption (2) Biodiversity (1) Emerging market multinational enterprises (1) Gainful employment (1) Gender (2) Global risk (1) Health (6) Justice (7) Mobile spatial data (2) Peace, justice, human rights Policy (1) Public institutions (3) Security (2) Social entrepreneurship (1) Supply chains (2)
‘SDG 16.6’ *Promote peaceful and inclusive societies for sustainable development, provide access to justice for all and build effective, accountable and inclusive institutions at all levels*	None	None
‘SDG’ + ‘Event management’	None	None
‘SDG’ + ‘Events management’	None	None
‘SDG’ + ‘Festivals’	14	Creation of shared value (1) Gender empowerment (1) Governance and policy (2) Heritage sites (1) Innovation and entrepreneurship (1) Social sustainability (1) Stakeholder benefits (1) Sustainable tourism (3) Transformation of events (1) Urban development (2)
‘SDG’ + ‘Occupational safety’	5	Health (1) Low carbon (1) OHS policies (1) Water safety (2)
‘SDG’ + ‘Workplace safety’	None	None
‘SDG’ + ‘OHS’ *Occupational health and safety*	5	Chemistry (1) Food safety (1) Health (2) Pollution (1)
‘SDG’ + ‘OSH’ *Occupational safety and health*	None	None
‘SDG’ + ‘Safety’	302	Aerospace (2) Agriculture (6) Alternative energy (15) Bio-economy (1) Circular economy (1) Climate change (7) Disaster risk (2) Economic growth (6) Education (8) Food safety and/or security (31) Gender inequality (8) Governance Green business (1) Healthcare (48) Migration (1) Occupational safety (6) Oceans (4) Primary caregivers (5) Recycling (1) Road safety (37) Social sustainability (4) Social-ecological system (1) Space (8) Sustainable chemistry (1) Sustainable engineering systems (1) Tourism corporate social responsibility (1) Urban development (12) Water safety and/or security (61)
‘SDG’ + ‘Sustainable tourism’	57	Carrying capacity (1) Children’s empowerment (1) Climate change awareness (4) Community tourism (3) Consumption behaviour (2) Critical discourse analysis (2) Economic growth (9) Ecotourism (4) Education (4) Employee behaviour (2) Gender (2) Global consciousness (1) Governance (5) Impactful research (1) Localisation (1) Revenue sharing (1) Spiritual tourism (1) Stakeholder engagement (7) Urban development (3) Water quality (1) Workplace dignity (1)

SDG, sustainable development goals; SME, small medium enterprises; OHS, occupational health and safety.

**FIGURE 1:**
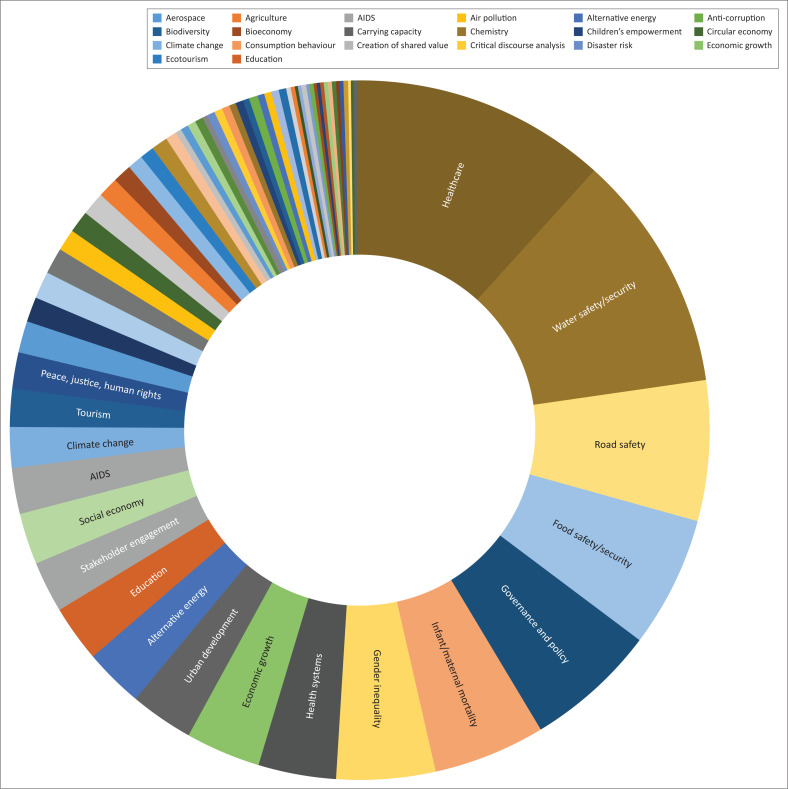
Main constructs from Scopus search.

The main constructs identified through the Scopus search (summarised in Table 1) can be illustrated as per Figure 1.

An analysis of these search results indicates that despite the emphasis placed on the SDGs by governments and higher educational institutions around the world, there is a distinct lack of academic publications on this topic. Furthermore, in the context of SDGs, the topics of both festivals and OSH are lagging even further behind other topics such as healthcare, water safety and security, road safety, food safety and security and governance. Occupational safety and tourism are amongst the lowest ranking results, with seven and 10 articles, respectively, despite the fact that this Scopus search specifically included these two terms.

One of the key goals of Agenda 2030 is the successful implementation of the Sendai Framework of Disaster Risk Reduction, holistically addressing risk management at all levels in all industries (UN 2015). The SDGs established by Agenda 2030 presents an opportunity to integrate the concepts of festivals and events with OSH. The importance of this integration hinges on these two industries both directly contributing to sustainable tourism and economic growth. These two industries are also deeply rooted in the mitigation of various types of risk. To this end, this article proposes that targeted aspects of the Sendai Framework be linked with the SDGs that relate to festivals and events management and OSH.

## Literature review

Occupational safety and health incidents are particularly high in developing nations, with the health of staff being a major determinant of productivity. Occupational safety and health is related to SDG dimensions related to substantial reductions in death and illnesses because of hazardous chemicals, protecting labour rights to promote safe and secure working environments, as well as transparent, effective and accountable institutions. Occupational safety and health gaps should thus be addressed to allow for a healthier workforce and increased productivity (ILO 2020a:1). Sustainable development goals comprise globally applicable goals aimed at balancing different dimensions of sustainable development, thus allowing the opportunity for South African businesses to apply inclusive processes aimed at addressing OSH challenges, which account for almost 9% of the South African gross domestic product (GDP). Synergies of health, sustainability and other goals can be utilised to allow for enhanced OSH (NIOH 2020:1).

Sustainable tourism encompasses the achievement of sustainable development by balancing environmental, economic and socio-cultural aspects. Tourism falls under accommodation and food service activities (Statistics South Africa 2012). This industry is committed to a low impact on the environment and can be used as a key driver for awareness and positive behavioural change amongst millions of people (UNWTO 2020:1). A situational analysis of the tourism industry has indicated that although the external environment of tourism is improving, more destinations are entering this competitive space with health and safety issues being highlighted as a key concern (Department of Tourism 2015:10).

### Overview of festival and events management

Festivals and events increase the visibility of host communities, thereby contributing to local economies. In the past, festivals were treated indistinctly from events, with festivals being defined as free and repeated festivities based on the commemoration of traditions. Events (which occurs at one point and might be a one-time occurrence), including festivals, should be properly managed to ensure benefits to society and to accurately determine its economic impact (Pereira et al. 2021). Therefore, festivals and events are often used as synonyms. The contribution of festivals and events to sustainability can be divided into three categories, in accordance with the pillars of sustainability, specifically the economy, environment and social community (Purvis, Mao & Robinson 2019). From a social perspective, festivals and events contribute to the host communities (Hazel & Mason 2020:181; Nordvall & Heldt 2017; Xie & Sinwald 2016) with its focus on culture, heritage and specific places (Black 2016). Related to the spaces and places where events take place, is the environmental aspect of sustainability. This aspect is addressed most succinctly by sustainable tourism that links in with the events taking place in a certain area (Liang et al. 2016; Moyle et al. 2020). Festivals and events also hold economic benefits for local business (Xie & Sinwald 2016).

Role players in events form part of a local organising committee (LoC), including event sponsors, event originators, event managers, event consultants, suppliers, media, accommodation providers, transport providers, retailers and general service providers (Dowson & Basset 2018; Getz & Page 2016). In addition, other stakeholders not necessarily presented on LoCs include customers, guests, participants, performers, officials and paid staff (Getz & Page 2016). Hazel and Mason (2020) proposed that information regarding the environmental sustainability should be communicated and that ensuring that attendees and other stakeholders are aware of the environmental sustainability values of an event, will encourage them to make decisions that are in line with these values. Events present a unique opportunity for the event stakeholders to directly impact aspects such as urban sustainability (Liang et al. 2016). This impact can be either positive or negative, necessitating the application of impact assessments on events to ensure that the economic gain is not emphasised at the cost of social and environmental impacts (Liang et al. 2016; Tanner et al. 2018).

### An overview of occupational safety and health in terms of disaster risk reduction

It is estimated that over 2.78 million people died in 2019 in OSH incidents across the world (ILO 2019; One Per Cent Safer Foundation 2020). Despite various interventions to address disasters and OSH incidences across many industries, this number is still unacceptably high. Even though it is not possible to fix everything at once, it is still possible to do something about this concerning trend. Despite the fact that this aspect is addressed as part of the SDGs, the systematic literature review conducted in this article points towards a lack of action and research in this regard. By working One Per Cent Safer, 28 000 more people will get home safely every year (One Per Cent Safer Foundation 2020). The model proposed in this article allows for continuous, incremental change. Changing one small thing at a time can make a huge difference for the festival and events industry and the surrounding community. Disaster risk reduction at this level, can only take place through the identification of hazards in communities, utilising risk management resources as efficiently as possible and actively managing risk perceptions (Becker, Hagelsteen & Abrahamsson 2021; Coetzee 2021; Odiase, Wilkinson & Neef 2020).

With 3.94% of the world’s annual GDP lost because of occupational related deaths and illness, it is clear that a more comprehensive approach to the management of OSH is required (ILO 2019). Incremental change can be achieved through the implementation of a multi-hazard impact-based early-warning system linked with the relevant SDGs (IRDR 2018; UN 2015). The elements of the multi-hazard impact-based early-warning system include institutional arrangement, data observation, data and information collection, hazard detection, hazard assessment, impact-based forecasting, infrastructure, notification, risk communication and connection and response (IRDR 2018).

Hazards, which are defined as a source of or exposure to danger (RSA 1993), poses risks to events and festival planners and managers (Bladen et al. 2018; Getz & Page 2016). Therefore, it is important that a proper risk assessment should be conducted to prevent or mitigate its impact on events and festivals (Bladen et al. 2018). Disaster risk reduction can only happen with proper risk assessment. To encourage event planners and managers to conduct risk assessments, they should be cognisant of the moral (preservation of life and quality of life), financial (budgeting for OSH) and legal (legislative directives) motivation behind risk assessment, especially whilst focusing on risk reduction (Esterhuyzen et al. 2019).

Risk management is a specialised skill within the festivals and events domain. Therefore, it is suggested to appoint a dedicated OSH executive, competent to conduct a risk assessment over the event or festival life cycle. As risk management is a specialised skill, events planners and managers (or other stakeholders) might not have the required credentials to conduct the assessment and more so a framework for guidance on how to interpret and apply the array of standards and legislations applicable to the industry. To complicate events risk more, events managers need to be familiar with the types of events risks, ranging from drug and alcohol abuse, terrorism, crime, fire safety, crowd management, recent COVID-19 protocols and future pandemic protocols (Bladen et al. 2018; Bowdin et al. 2011; Getz & Page 2016). According to section 24 of the Bill of Rights in the Constitution of South Africa (RSA 1996) *everyone has a right to an environment that is not detrimental to their health or well-being*. The right to an environment that is not detrimental to their health and well-being as per the constitution, links directly to SDG 3 – good health and well-being.

In the *South African Occupational Health and Safety Act* (85 of 1993), risk is defined as the probability that injury or damage will occur. The International Organization for Standardization (ISO) adds to this definition by including the effect of uncertainty. When evaluating the risks present in an event or festival, the likelihood of the risk occurring should be investigated. Likelihood is the chance of something happening. According to ISO (2018:1) risk management is the coordinated activities that are aimed at directing and controlling an event or festival in terms of the risks faced.

**FIGURE 2:**
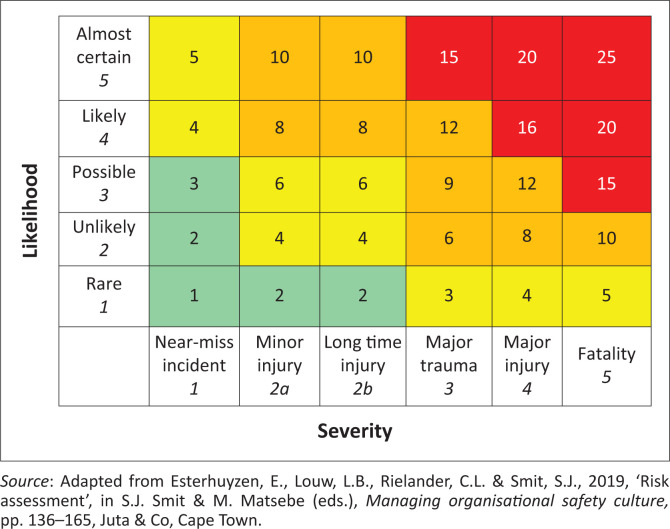
Matrix of likelihood of occurrences and severity of loss.

During the risk assessment process, it is necessary to determine how serious the consequences of an event risk can be and the likelihood of the incident to occur. Five levels are used to the measure the likelihood that an incident can occur, as indicated in Figure 2, where Level 1 indicates the least likely occurrence and Level 5 the most likely occurrence. Incident impacts (or the severity of loss) are also classified according to levels, where Level 1 indicates the insignificance of the incident and Level 5 the catastrophe as a result of the incident. Ideally these risk incident impacts can be plotted on a events/festival risk analyses matrix (Esterhuyzen et al. 2019:160–161; Van der Wagen & White 2010).

During the risk evaluation phase, the event or festival manager needs to decide what action should be taken to address the identified risk. This could include doing nothing, considering the possible risk treatment options, conducting additional risk analysis to gather further information, maintaining the existing risk control measures or reconsidering the objectives of the event and subsequent risk assessment process. Risks that fall within the red and orange categories of Table 1 should be addressed as a matter of urgency and prioritised. Risk treatment is informed by the risk assessment that has been conducted. Risk treatment options should assist the organisation in avoiding the risk by excluding an activity, removing the source of the risk, changing the likelihood of occurrence, changing the consequences of a risk event, sharing the risk (insurance) or making an informed decision to retain the risk and keep the status quo. It is also possible to increase the risk in order to pursue a specific opportunity (ISO 2018:13).

#### Institutional arrangement

**FIGURE 3:**
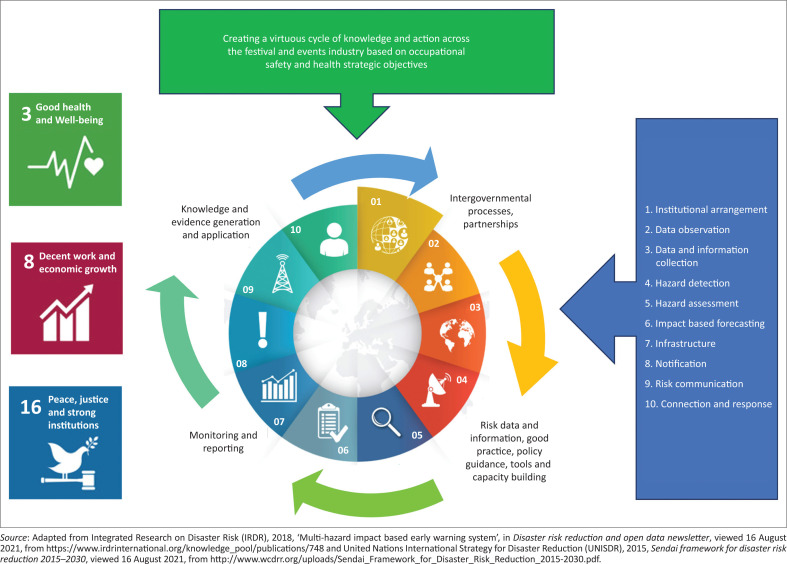
Proposed framework to enhance the sustainable development goals application for disaster risk reduction in the festivals and events industry based on strategic OHS objectives.

In this aspect of the proposed framework illustrated in Figure 3, it is important to take cognisance of the regulatory framework, roles and responsibilities, interagency collaboration and strategic operational objectives (IRDR 2018). Sustainable development goals 16 focuses on promoting peaceful and inclusive societies for sustainable development, providing access to justice for all and building effective, accountable and inclusive institutions at all levels (UN 2015). This focus on institutional arrangement addresses the fundamental human rights and human dignity of all people around the world (UN 2020). These basic human rights extend into the working environment, with the ILO placing special emphasis on the prevention of workplace accidents and death in all industries. An unsafe workplace not only endangers human rights in the workplace but severely impacts the surrounding communities by directly contributing to unemployment (ILO 2009).

#### Data observation

Continuous observation is required in terms of OSH and data observation is included in the proposed framework to enhance the sustainable development goals application for disaster risk reduction. At the heart of implementing a healthy safety culture in any organisation is observation. Observation is also a core component of proactive behaviour-based safety (PBBS). In short, PBBS is a method of modifying unsafe behaviour in the workplace (Li et al. 2015). Regular observation of both document and human data creates an opportunity for on-the-spot correction of at-risk behaviour (Lal 2019). The sharing of these observations is discussed later in the proposed model.

The SDG 3, ensuring healthy lives and promoting well-being for all at all ages, directly relates to the goals of both data observation and PBBS by ensuring that the number of deaths and illness from hazardous chemicals, pollution and contamination (SDG 3.9) is addressed in workplaces (UN 2015).

#### Data and information collection

All applicable sources of information, including contemporary publications by the ILO, the UNWTO, the NIOH, as well as academic, peer reviewed articles were consulted in an effort to make informed decisions on OSH. Safety information as observed in the previous step was collected and collated in preparation for the development of the proposed model to inform hazard and risk assessments and to form the basis of safety training (Lal 2019).

#### Hazard detection

In terms of the proposed model, hazard identification needs to move beyond the assessment of elements that adhere to the functional and structural characteristics of hazards (Esterhuyzen & Louw 2019a) that could pose a danger to attendees, to also include aspects that threaten the lives and safety of staff. Traditional events risk management models focus on the visible hazards at an event or festival, which can be observed in a walk-through (Dowson & Bassett 2018).

Hazard detection and identification should be based on a science-based approach, determining the characteristics of hazards, in order to establish what the extent of the interaction between them would be and what the resulting risk is likely to be (Esterhuyzen & Louw 2019b). The characteristics of hazards can be divided into structural and functioning characteristics. The structural characteristics of hazards are tangibility, density, size, weight, shape and texture whilst the functioning characteristics relate to energy, consistency and interaction (Esterhuyzen & Louw 2019a).

This phase of the proposed framework directly contributes to Goal 3 of Agenda 2030, by facilitating the detection of hazards that threaten the health and well-being of people at work. A specific focus on target 3.9 is achieved by detecting the hazards that can be addressed to reduce the number of deaths and illnesses from hazardous chemicals, pollution and contamination (UN 2015).

#### Hazard assessment

The elements of this phase of the proposed model consist of observation, the application of PBBS, conducting risk assessment and managing risks through avoidance, mitigation, elimination or transference (Bahr 2015). Risk management is both a strategic-level and an operations-level activity, including both generic risks and risks that are specific to the industry or specific event (Evans 2020). Risks should be measured against the possible gains in order to determine whether it would be acceptable to continue with an event, weighing the potential gains against possible losses. Social spaces such as festivals and events are characterised by less coherent and quantifiable anxieties and fears than one would encounter at a technical or financial level (Clarke 2018).

#### Impact-based forecasting

Impact-based forecasting is informed by hazard assessment and constitutes an impact and risk assessment of a specific festival or event. At the heart of impact-based forecasting is risk management. Strategic planning and risk analysis should be conducted for the events management business as a whole and also for each separate event project that this business becomes involved in (Getz & Page 2016). These evaluations can be conducted as a cost-benefit analysis, impact analysis, sensitivity analysis or scenario planning (Evans 2020). Any one or combination of these non-financial risk analysis tools can be utilised to identify the risks posed to the business, staff, customers, attendees and other stakeholders as part of a sustainable governance process.

#### Infrastructure

Goal 16 as outlined in Agenda 2030 highlights the promotion of peaceful and inclusive societies for sustainable development, providing access to justice for all and building effective, accountable and inclusive institutions at all levels (UN 2015). With specific reference to target 16.6 (developing effective, accountable and transparent institutions at all levels), the proposed framework relies on the principles of safety engineering to ensure the placement and selection of infrastructure that will address the aims of Goal 16 as outlined here, whilst simultaneously addressing the identified hazards and risks. In this context safety engineering is concerned with utilising design and engineering principles in a manner that addresses safety concerns. The elements of safety engineering would include subsets such as safety culture, system safety processes, risk management processes, all of which are utilised to achieve the goals of safety engineering (Bahr 2015; Mroszczyk 2012).

#### Notification

A safety culture as dictated by PBBS relies on open communication and stakeholder engagement (ed. Mochuana 2020). Safety training should, in the first place, address any safety transgressions observed during the data observation phase. This is achieved through regular tool-box talks and other safety training interventions (Lal 2019). However, the festival and events management business’s responsibility does not end there. A truly successful safety culture will endeavour to train stakeholders, including customers and attendees, in safe, sustainable practices as well, making training available at different levels as applicable (Li et al. 2015).

Specialised notification networks should be in place to ensure that the correct information is disseminated through social media and the internet (IRDR 2018). Information circulates quickly and people have multiple sources of information that can be consulted at a moment’s notice (Bieder 2018). To this end, specialised notification networks should endeavour to counter false news and communication and adapt continuously to the needs of the organisation whilst responding to changing safety needs.

#### Risk communication

The context of risk communication will differ between situations and events (Bastide 2018). Although a focus on individual safety is paramount to address OSH, the scope of communication needs to be broader to ensure safety at a local community level and inform policy changes (Bieder 2018). A broader scope of safety communication would involve the notification of governments and the broader public (IRDR 2018). Only through influencing policies at a government and public level will the major issues of OSH be addressed. This ties in directly with Goal 16 and specifically targets 16.6 again, influencing institutions at all levels (UN 2015). It is advisable to have a risk communication policy in place before it is required by a change in circumstances that would lead to significant changes in risk (Bieder 2018).

#### Connection and response

When the scope of communication is broadened to include the public and the government, it should be observed that each of these stakeholders have multiple and often contrasting interests that could cause tension (Bieder 2018). In order to address and mitigate these contrasting interests and resulting tension, emphasis should be placed on reciprocal communication.

In the process of responding to the information gathered and assessments conducted in the earlier steps of this model, it is important to ensure the implementation of proactive preventative measures. Proactive preventative measures form a key part of PBBS and the effectiveness thereof can be measured through a Safety Index (Li et al. 2015). Risk perception, knowledge and interpretation also inform this final part of the proposed framework (IRDR 2018), measuring public awareness, the successful implementation of risk management and risk mitigation procedure.

One of the measures that can be used to determine the successful implementation of the proposed framework is to look at the attitudes towards safety. Successfully addressing OSH issues, improving a safety culture and training both staff and stakeholders should be evident in changed attitudes towards safety (Mroszczyk 2012).

In the 2020 SDG report, the plight of workers around the world is highlighted, specifically in terms of the threats to their occupational health and safety. This report on SDG 8 indicates a decline in economic growth even before the COVID-19 pandemic gripped the world. With 10 work-related fatalities out of every 100 000 workers, the statistics are not improving in the climate of economic decline as occupational safety takes a back-seat to other economic priorities in the workplace (UN 2020). The role of governments and policymakers is highlighted as a required support for businesses to address the OSH challenges they face (UN 2020).

The disaster risk reduction checklist for the festival and events industry section illustrates the proposed framework to enhance SDG application in the festivals and events industry based on strategic OSH objectives. This framework combines the application of SDGs, the elements of the multi-hazard impact-based early-warning system and the Sendai Framework for disaster risk reduction.

### Proposed framework for event managers to consider in event management

Figure 3 is informed by the SDGs, the elements of the multi-hazard impact-based early-warning system and the Sendai Framework for disaster risk reduction. This figure illustrates the recommended interaction between SDGs, strategic OSH objectives and their application for consideration within the festival and events industry. This proactive approach aims to assist in the reduction of OSH incidents within the festival and events industry whilst promoting the achievement of the Agenda 2030 SDGs.

The process of implementing this model for disaster risk reduction in the festival and events management industries should ensure the inclusion of all stakeholders. To this end, festival and event managers and businesses are responsible for the education of their staff, clients and attendees regarding safe, sustainable practices.

### Disaster risk reduction checklist for the festival and events industry

Table 2 proposes a disaster risk reduction checklist for the festival and events industry for consideration in order to contribute towards the achievement of the relevant SDGs.

**TABLE 2:** Disaster risk reduction checklist in the festival and events industry.

Variables	Checks
Strategy 1: Institutional arrangements	□ Regulatory framework □ Mandate of event □ Roles and responsibilities of festival stakeholders □ Enhance collaboration between festival stakeholders □ Clear project outline with concept of operation
Strategy 2: Data observation	□ Environmental impact assessment □ Urban environment assessment □ Potential impact of natural / weather phenomenon
Strategy 3: Data and information collection	□ National and local safety data □ Global safety data □ National and local risk management directives □ Intergovernmental processes and partnerships
Strategy 4: Hazard detection	□ Identify all hazards using functioning and structural characteristics of hazards □ Hazard detection systems □ Risk identification □ Evaluate available risk data □ Identify good practice □ Identify policy and procedural guidelines
Strategy 5: Hazard assessment	□ Hazard observation informed by stakeholders □ Hazard assessment criteria for the event □ Disaster and risk prediction models □ Uncertainty assessments around specific event
Strategy 6: Impact-based forecasting	□ Informed by hazard assessment □ Identify vulnerabilities of events □ Disaster risk impact assessment
Strategy 7: Infrastructure	□ Enhance economic contribution made by the event by contributing to job creation in local community □ Implement risk mitigation strategies when constructing permanent and temporary infrastructure □ Temporary infrastructure for crowd management □ Installation of infrastructure on access ways and roads to enhance safety
Strategy 8: Notification	□ Monitoring and reporting of event risk □ Continuous engagement with internal and external stakeholders □ Incorporate disaster risk reduction communication on social media platforms for the event
Strategy 9: Risk communication	□ Risk communication with national and local authorities □ Continuous engagement between local organising committee and affected communities □ Enhanced tourist communication
Strategy 10: Connection and response	□ Contribute to the existing body of knowledge on disaster risk reduction □ Evidence generation for informed event risk response □ Pre-event risk mitigation strategies □ Integrating local knowledge and expertise □ Improved public awareness □ Event risk perception, knowledge and interpretation □ Communication of appropriate risk responses already in place

## Implication and conclusion

Sustainable Development Goals are aimed at achieving a sustainable future for all. The detrimental effect of OSH incidents can be counterproductive to achieving such goals and should therefore be closely monitored and managed through active disaster risk reduction methods. Festival and event managers and stakeholders should thus take cognisance of the importance of OSH through a plan of action, benchmarked against best practice, to allow for a sustainable future for all stakeholders. The likelihood and severity of loss associated with event and festival risks should thus be properly understood by all stakeholders to allow for sustainable risk management practices within the industry, aimed as sustainability.

Future research should be aimed at exploring SDGs in festivals, tourism and OSH further in order to contribute to an academically sound body of knowledge. It is also recommended that the theoretical framework developed in this article be implemented and the success of such implementation thoroughly researched and documented. It is suggested that a checklist be developed aimed at properly integrating OSH, SDGs and event risk management.
